# Knee injury mechanism varies by injury category: Video analysis of multi‐ligament knee injuries in the National Football League

**DOI:** 10.1002/ksa.70025

**Published:** 2025-09-04

**Authors:** Ethan Ruh, Tyler Kallman, Elizabeth Lyden, Justin Greiner

**Affiliations:** ^1^ College of Medicine University of Nebraska Medical Center Omaha Nebraska USA; ^2^ Department of Orthopaedic Surgery University of Nebraska Medical Center Omaha Nebraska USA; ^3^ College of Public Health University of Nebraska Medical Center Omaha Nebraska USA

**Keywords:** American Football, arthroscopy, knee, ligament, sports medicine

## Abstract

**Purpose:**

This study aims to characterize the mechanism of multi‐ligament knee injury (MLKI) sustained during a National Football League (NFL) game through video analysis.

**Methods:**

A retrospective video analysis of official NFL game footage spanning 1997–2022 was performed. Players with MLKIs were identified from publicly available injury surveillance data. Player and game demographic information was collected. Athletes were categorized based on ligament injury patterns. Videos including MLKI were analyzed to categorize injuries based on non‐contact, indirect or direct contact mechanisms and determine the hip, knee and ankle position at the time of injury. Differences across MLKI groups were identified by Fisher's exact test.

**Results:**

Thirty‐five MLKIs were identified. MLKIs most commonly occurred from direct contact to the limb (80%), with the second most common injury mechanism being indirect contact (14.3%). The most common MLKI was injury to the anterior cruciate ligament (ACL) and medial collateral ligament (MCL) (65%), while the second most common was the ACL and lateral collateral ligament (LCL) (15%). Direct contact MLKI most often resulted in a valgus and external rotational force about a flexed knee (58%). There were statistically significant differences when comparing the direction of contact, hip and knee position between injury MLKI groups. Combined ACL and MCL injury commonly occurs with knee flexion, valgus and external rotation. Combined ACL/posterior cruciate ligament (PCL) or ACL/LCL injuries occurred most commonly with varus and internal rotational forces on an extended knee. Multiligament injuries involving three ligaments occurred only from direct contact mechanisms.

**Conclusion:**

Various injury mechanisms and characteristics result in different types of MLKI in NFL athletes, though the vast majority of MLKIs occur from direct contact forces to the knee. Combined ACL and MCL injury occurred most frequently, resembling the mechanism and lower extremity position for isolated ACL injury, though it frequently occurs due to direct contact with the knee.

**Level of Evidence:**

Level IV.

**Abbreviations**
ACL: anterior cruciate ligament
ESPN: Entertainment and Sports Programming Network
LCL: lateral collateral ligament
MCL: medial collateral ligament
MLKI: multi‐ligament knee injury
NFL: National Football League
PCL: posterior cruciate ligament

## INTRODUCTION

The occurrence of injuries is higher in the National Football League (NFL) than in any other professional sport [3, 10]. Across 2012–2016, it was found that the most common site of injury was the knee, which accounted for 17.2% of injuries, while the ankle and back accounted for 13.6% and 8.8%, respectively [2]. The most common and well‐researched knee ligament injury, the tear of the anterior cruciate ligament (ACL), is a season‐ending injury and causes a significant decline in games played and game performance [11].

Although less common, multi‐ligament knee injury (MLKI)—which involves tears of more than one ligament in the knee—is more severe. These injuries can significantly impact an athlete's career and longevity, as shown in a review of fifty NFL athletes with MLKIs, reporting an overall return‐to‐play rate of 64% [1]. In NFL athletes, those who sustained MLKIs, involving the ACL and medial collateral ligament (MCL), had a return‐to‐sport rate of 70.8%, with a 43.5% chance of returning to their original level of play. In contrast, athletes with MLKIs involving the lateral collateral ligament (LCL) or posterior cruciate ligament (PCL) had a lower return‐to‐sport rate of 55.6%, and only an 18.5% chance of returning to their original level of play [1]. The disparity between the return‐to‐sport and return to the previous level of play highlights the career‐altering nature of these injuries, given that a player may return to sport but participate in far fewer snaps and start fewer games. Further, previous studies have found that combined ACL and MCL injuries are more common and believed to be of lower severity than MLKIs involving the PCL or posterolateral corner [1, 6, 7].

Previous research utilizing video analysis on ACL injuries in NFL players found that over 70% occurred in non‐contact or indirect contact mechanisms, while also identifying that most non‐contact ACL injuries occurred with the knee in flexion and valgus, the ankle abducted and externally rotated, and the hip abducted and flexed [9]. However, video analysis of MLKIs has been limited, and MLKIs are far less understood than isolated ACL injuries. To date, there has not been a demographic and video analysis of MLKIs in professional American Football athletes. Therefore, the purpose of this study was to characterize MLKIs in NFL athletes while including video analysis to describe injury mechanism and characteristics, including lower extremity position at the time of injury. We hypothesized that the majority of MLKI injuries would occur secondary to direct contact, and the lower extremity position would differ between the different MLKI patterns.

## METHODS

### Data collection

Players with MLKIs sustained between the Years 1997 and 2022 were identified from publicly available NFL injury surveillance data and confirmed via separate web searches of official team reports or Entertainment and Sports Programming Network (ESPN) news articles, primarily through internet search of terms such as ‘NFL multi‐ligament knee injury’, ‘football multi‐ligament knee injury’ as well as injuries highlighted through the X account ‘ACL Rehab Club’. Following the identification of an injured player, demographic information and injury information were obtained to include position, height, weight, team, order in‐depth chart, week of season, quarter of game, playing surface and playing conditions. Information regarding when the injury occurred was obtained and used to identify the video clip of the injury. Athletes were placed into categories depending on the injury pattern, which is based on previous research on knee dislocation injuries (Table 1) [10, 15]. Combined ACL/MCL injuries were categorized as Group I. Two ligament injuries not involving the MCL were categorized as Groups IIa and IIb. Three ligament injuries were categorized as Categories IIIa and IIIb [1, 12, 15].

**Table 1:** Classification of multi‐ligament knee injuries (MLKIs).

Classification of MLKIs	
I	ACL + MCL
IIa	ACL + LCL
IIb	ACL + PCL
IIIa	ACL + PCL + MCL
IIIb	ACL + LCL + PCL/PLC

*Note*: Adapted from Anatomic Classification of Knee Dislocation and recategorized based upon previous research on MLKIs [15].

Abbreviations: ACL, anterior cruciate ligament; LCL, lateral collateral ligament; MCL, medial collateral ligament; PCL, posterior cruciate ligament.

Video clips were obtained from NFL+, a streaming service available through NFL.com that contains complete playback of any professional game played between 2009 and the present. For athletes who sustained injuries before 2009, internet searches were conducted to find as many clips as possible of the injury. Inclusion criteria for the study were an NFL athlete sustaining an MLKI with adequate video footage to allow video analysis and characterization of the knee injury. Athletes with isolated, single ligament injuries were excluded. Players who sustained an MLKI in practice or in another environment in which video of the injury was unavailable were excluded from video analysis but included in the initial analysis of demographic associations with MLKI. Five‐second video clips of the MLKI injury were created using the screen recording feature of Apple macOS Mojave. Video clips included player activity before the injury, the moment of injury, and shortly following the injury. If multiple angles were provided from official replay, these were also recorded in 5‐s clips and compiled.

### Video analysis

**Figure 1:**
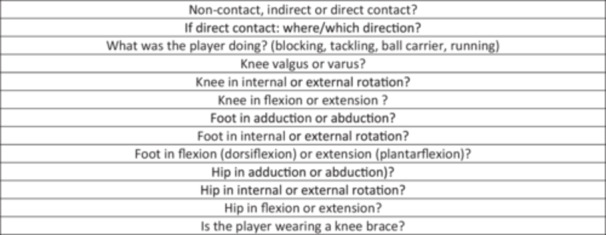
The questionnaire used for video analysis of NFL knee injuries. This figure illustrates the standardized questionnaire designed to assess lower extremity joint positioning during injury events captured on video. Two authors used the questionnaire to document the position and motion of the knee, hip, and ankle at the moment of injury. NFL, National Football League.

To analyze video clips, a nine‐question questionnaire was created to assess each video clip, assessing flexion/extension, adduction/abduction and internal/external rotation about the hip, knee and ankle (Figure 1). Two of the authors (an orthopaedic surgeon with a fellowship in sports medicine and a medical student) analyzed the video clips individually. The two score sheets were compared after scoring, and disagreements in scoring were discussed together to reach a consensus. During video analysis, injuries were classified as direct contact, indirect contact or non‐contact injury. Direct contact injury was defined as direct contact to the injured knee, while indirect contact was classified as a contact injury that occurred without direct contact to the affected player's knee.

### Statistical analysis

Descriptive statistics (means, standard deviations, medians, counts and percentages) were used to summarize the data. Analysis of variance was used to compare continuous variables, while Fisher's exact test was used for categorical variables when comparing player and game demographics with injury groups. All analyses were done using SAS, Version 9.4. A *p*‐value < 0.05 was considered statistically significant.

## RESULTS

### Demographics

Forty multi‐ligament injuries were identified after initial search. Of these, 35 players were found to have video clips eligible for analysis, with 5 excluded due to injury occurring in practice. Of the 35 players, 31 (88.6%) had multiple angles of video footage of the injury, while only 4 (11.4%) players had only one angle of footage. Injuries that had only one angle occurred prior to 2014.

MLKI occurred throughout the season, with a similar distribution of the first half of the season and the second half of the season (Table 2). MLKI most often occurred during the first (37%) and third (34%) quarters of games. MLKIs occurred on turf in 23 (57.5%) injuries and on grass in 17 (42.5%) injuries. All observed MLKI injuries occurred in dry weather conditions, with none occurring during wet or rainy weather.

**Table 2:** Distribution of injury throughout season.

Time in season	Count
Preseason	6 (17.1%)
Early season (Weeks 1–8)	13 (32.5%)
Late season (9–17)	13 (32.5%)
Playoffs	3 (8.6%)

*Note*: Data presented as number (percentage of total).

Players who sustained an MLKI had a mean BMI of 30.75 ± 4.15. Thirty players who sustained MLKI were starters (85.7%). Offensive skill positions, including quarterbacks (*n* = 7, 17.5%) and wide receivers (*n* = 7, 17.5%), had the highest rates of sustaining an MLKI (Table 3). Twenty (57.1%) athletes sustained MLKI while running as the ball carrier, which was the most common activity during MLKI (Table 4). Thirty‐three (97.1%) athletes were not wearing a knee brace at the time of injury.

**Table 3:** Position of players who suffered multi‐ligament knee injury.

Position	Count
Quarterback	7 (17.5%)
Wide receiver	7 (17.5%)
Tight end	6 (15.0%)
Running back	5 (12.5%)
Centre	4 (10.0%)
Offensive guard	3 (7.5%)
Offensive tackle	1 (2.5%)
Linebacker	3 (7.5%)
Cornerback	2 (5.0%)
Safety	1 (2.5%)
Defensive tackle	1 (2.5%)

*Note*: Data presented as number (percentage of total).

**Table 4:** Activity of players who suffered multi‐ligament knee injury.

Player activity	Count
Ball‐carrier	20 (57.1%)
Blocking	6 (17.1%)
Throwing	3 (8.6%)
Catching	2 (5.7%)
Open‐field running	2 (5.7%)
Tackling	2 (5.7%)
Missing	5

*Note*: Data presented as number (percentage of total).

### Video analysis

Initial inter‐rater agreement among the authors conducting the video analysis was 76%. The most frequent source of disagreement pertained to the positional assessment of the foot, accounting for 54% of all discrepancies. These disagreements primarily occurred when one author deemed the joint position indeterminable based on the video footage, whereas the other considered it discernible. Upon joint review of all video clips, consensus was achieved in 100% of cases.

### All MLKI

At the time of MLKI, there was direct contact to the knee in 28 (80%), indirect contact in 5 (14.3%), with 2 (5.7%) MLKI occurring from non‐contact mechanisms. Direct contact MLKIs most frequently occurred with a lateral force to the knee (*n* = 19, 67.9%). Across all MLKI categories, the most common position of the knee was valgus (*n* = 29, 85%), external rotation of the tibia (*n* = 25, 73.5%) and knee flexion (*n* = 24, 70.6%). The most common position of the ankle during injury was eversion (*n* = 25, 78%), external rotation (*n* = 26, 81%) and dorsiflexion (*n* = 24, 70.0%). The most common position of the hip during injury was adduction (*n* = 31, 91.0%), internal rotation 28 (82.0%) and flexion (*n* = 30, 88.2%) (Table 5). Video analysis results varied across MLKI categories regarding direction of impact, knee valgus/varus, knee rotation, knee flexion/extension and hip rotation.

**Table 5:** Video analysis classified by injury category.

Parameter	Categorization	Group 1, ACL + MCL (65%)	Group 2, ACL + LCL (15%), ACL + PCL (5%)	Group 3, ACL + LCL + PCL (7.5%), ACL + MCL + PCL (7.5%)	*p*
Direction of contact	Anterior/anteromedial	0 (0.0%)	2 (50.0%)	0 (0.0%)	0.009
	Lateral/posterolateral	16 (84.2%)	2 (50.0%)	2 (40.0%)	
	Anterolateral	3 (15.8%)	0 (0.0%)	3 (60.0%)	
	Missing	7	4	1	
Location of contact	Above knee	1 (5.3%)	1 (25.0%)	0 (0.0%)	0.218
	At knee	17 (89.5%)	2 (50.0%)	5 (100.0%)	
	Below knee	1 (5.3%)	1 (25.0%)	0 (0.0%)	
	Missing	7	4	1	
Knee valgus/varus	Valgus	23 (100%)	2 (33.3%)	4 (80.0%)	<0.001
	Varus	0 (0.0%)	4 (66.7%)	1 (20.0%)	
	Missing	3	2	1	
Knee rotation	Internal rotation	3 (13.6%)	4 (66.7%)	3 (60.0%)	0.002
	External rotation	19 (86.4%)	2 (33.3%)	2 (40.0%)	
	Missing	3	2	1	
Knee flexion/extension	Flexion	19 (82.6%)	1 (16.7%)	4 (80.0%)	0.009
	Extension	4 (17.4%)	5 (83.3%)	1 (20.0%)	
	Missing	3	2	1	
Ankle adduction/abduction	Adduction	3 (13.6%)	3 (50.0%)	1 (25.0%)	0.082
	Abduction	19 (86.4%)	3 (50.0%)	3 (75.0%)	
	Missing	4	2	2	
Ankle rotation	Internal rotation	3 (13.6%)	3 (50.0%)	0 (0.0%)	0.101
	External rotation	19 (86.4%)	3 (50.0%)	4 (100.0%)	
	Missing	4	2	2	
Ankle flexion/extension	Flexion	20 (90.9%)	4 (66.7%)	5 (100.0%)	0.238
	Extension	2 (9.1%)	2 (33.3%)	0 (0.0%)	
	Missing	4	2	1	
Hip adduction/abduction	Adduction	21 (91.3%)	5 (83.3%)	5 (100.0%)	0.704
	Abduction	2 (8.7%)	1 (16.7%)	0 (0.0%)	
	Missing	3	2	1	
Hip rotation	Internal	21 (91.3%)	2 (33.3%)	5 (100%)	0.009
	External	2 (8.7%)	4 (66.7%)	0 (0.0%)	
	Missing	3	2	1	
Hip flexion/extension	Flexion	22 (95.7%)	4 (66.7%)	4 (80.0%)	0.089
	Extension	1 (4.3%)	2 (33.3%)	1 (20.0%)	
	Missing	3	2	1	

*Note*: Data presented as count (percentage of total). Missing included those injuries that occurred in practice and those for which video coverage was not sufficient to accurately assess the variable.

Abbreviations: ACL, anterior cruciate ligament; LCL, lateral collateral ligament; MCL, medial collateral ligament; PCL, posterior cruciate ligament.

### Category I injury

Category I injury (ACL and MCL) was the most common MLKI, occurring in 26 (65.0%) of injuries. There were 19 (82.6%) of Category I injuries occurring from direct contact, 3 (13.0%) from indirect contact, and 1 (4.3%) from a non‐contact mechanism. Direct contact Category I MLKIs most frequently occurred with a lateral or posterolateral force to the knee (*n* = 16, 84.2). The most common position of the knee was valgus (*n* = 23, 100.0%), external rotation of the tibia (*n* = 21, 91.3%) and knee flexion (*n* = 19, 82.6%). The most common position of the ankle during injury was abduction (*n* = 19, 86.4%), external rotation (*n* = 19, 86.4%) and flexion (*n* = 20, 90.9%). The most common position of the hip during injury was adduction (*n* = 21, 91.3%), internal rotation (*n* = 28, 82.0%) and flexion (*n* = 22, 95.7%). All direct contact Category I injuries that occurred resulted in valgus force at the knee. Twenty‐one (91.3%) Category I injuries occurred with external rotation of the tibia, while two (8.7%) occurred with internal rotation of the tibia. Nineteen (91.4%) of Category I injuries occurred with the knee in flexion, while four (8.7%) occurred with the knee in extension. Twenty‐two (95.7%) of Category I injuries occurred with internal rotation of the hip, and one (4.3%) injury occurred with external rotation of the hip.

### Category II injury

Category IIa injuries (ACL and LCL) were the second most common MLKI, occurring in six (15%) athletes. Category IIb injuries (ACL and PCL) were the least common MLKI, occurring in two (5.0%) athletes. Four (50.0%) of Category II injuries occurred from direct contact, two (25.0%) occurred from indirect contact, one (12.5%) occurred from non‐contact mechanism, and one (12.5%) did not have video footage for analysis. Direct contact Category II injuries occurred equally from lateral/posterolateral and anterior/anteromedial forces (both *n* = 2, 50.0%). The most common position of the knee was varus (*n* = 4, 66.7%), internal rotation of the tibia (*n* = 4, 66.7%) and knee extension (*n* = 5, 83.3%). The most common position of the ankle during injury was flexion (*n* = 4, 66.7%), with the foot in adduction/abduction and experiencing equal internal and external rotation. The most common position of the hip during injury was adduction (*n* = 5, 83.3%), external rotation (*n* = 4, 66.7%) and flexion (*n* = 4, 66.7%).

### Category III injury

Category IIIa and IIIb injuries (ACL + PCL + MCL or ACL + PCL + LCL) were the third most common injury category, both occurring three times (7.5%). One Category III injury was excluded, as no video footage was available. All Category III injuries occurred from direct contact. Three (60.0%) direct contact Category III injuries occurred from anterolateral contact, and two (40.0%) occurred from lateral/posterolateral contact. The most common position of the knee was valgus (*n* = 4, 80.0%), internal rotation of the tibia (*n* = 3, 60.0%) and knee flexion (*n* = 4, 80.0%). The most common position of the ankle during injury was abduction (*n* = 3, 75.0%), external rotation (*n* = 4, 100%) and flexion (*n* = 5, 100%). The most common position of the hip during injury was adduction (*n* = 5, 100.0%), internal rotation (*n* = 5, 100.0%) and flexion (*n* = 4, 80.0%).

## DISCUSSION

The most important findings of this study were that MLKI most often occurs from direct contact and that the injury mechanism varies by injury categorization. In addition to confirming the heterogeneity of MLKIs, the study identifies specific player demographics most at risk for these injuries, addressing a previously underexplored area. Notably, it establishes that MLKIs most commonly result from direct contact forces during tackles, particularly involving ball carriers, and that combined ACL and MCL injuries are the most frequent injury pattern. Furthermore, this study adds novel biomechanical insights by describing differences across injury categories for direction of contact, degree of knee flexion/extension, knee valgus/varus alignment, knee rotation and hip rotation at the moment of injury—details that can inform future prevention and rehabilitation strategies.

Previous research on NFL athletes found that isolated ACL injury most frequently occurred while pursuing the ball carrier and performing a lateral cutting movement as a defensive back, or via direct contact to the knee as an offensive lineman [4, 9]. This study uniquely identifies offensive skill position players—specifically quarterbacks, wide receivers, running backs and tight ends—as being disproportionately affected by MLKIs in the NFL. Video analysis revealed that these injuries most commonly occurred during ball‐carrying plays, often following high‐velocity, direct‐contact tackles in which a defensive player delivered a diving blow to the knee. Although not formally quantified, this recurring mechanism highlights a critical positional vulnerability that has not been previously emphasized in the literature. These findings not only advance the understanding of MLKI epidemiology but also underscore the need for targeted prevention strategies, potential rule modifications to better safeguard offensive players, and position‐specific considerations in return‐to‐play protocols and long‐term risk assessments.

Injuries to defensive players were rare, occurring in only seven instances across the cohort. When they did occur, they were typically sustained while pursuing the ball carrier, most often resulting from being blocked, such as through cut‐blocks in the open field, or from falling onto the lower extremity during a pass rush. This pattern contrasts sharply with the mechanisms observed in offensive players, who were far more likely to sustain MLKI through direct, high‐velocity contact. The relative infrequency and differing mechanisms of defensive player injuries further emphasize that offensive athletes are at greater risk for MLKIs, reinforcing the need for position‐specific prevention strategies and rule considerations aimed at reducing these high‐impact collisions.

Analysis of the setting in which MLKI occurred demonstrated a similar distribution between the first and second halves of the season. However, 25 (71.4%) occurred in the first or third quarters of gameplay. To compare the results of the current study, prior evaluation of isolated ACL injury in professional athletes did not identify an association with an injury during the time of game or time of the season [16]. Further, the current study found MLKI occurred on grass and artificial turf in 42.5% and 57.5% of injuries, respectively. However, this study did not evaluate the number of exposures to artificial grass or turf to determine the risk per exposure. Previous research investigating lower extremity injury in NFL athletes from 2000 to 2009 found that the observed rate of ACL injury and eversion ankle sprains were 67% and 31% higher on artificial turf, respectively [8]. Finally, all observed MLKI occurred during dry weather conditions, indicating the potential influence of friction between the shoe and the ground in MLKI.

Category I injuries, or combined ACL and MCL knee injuries, were the most common injury pattern observed in the current study. ACL/MCL injury most often occurs from direct contact to the lateral knee, which imposes a valgus and external rotational force about a flexed knee, as well as hip internal rotation. This most closely emulates the ‘position of risk’ identified with the hip in flexion and abduction, the knee in early flexion, and the ankle abducted and externally rotated [9]. These results are not limited to American football athletes; previous video analysis of ACL injuries in professional Italian soccer players found that direct contact ACL injury occurred from lateral force applied to a valgus knee, and indirect and non‐contact injury occurred with dynamic knee valgus, abducted hip and a flat, externally rotated ankle [5]. The similarly identified position of risk may be an important predisposing factor to both injury types, with the added energy of high‐velocity direct contact being the important factor for determining the extent of ligamentous damage. Previous research on return‐to‐play and return‐to‐performance found that combined ACL and MCL injury had more favourable prognoses than other MLKI [1]. Combined ACL/MCL injuries are significant injuries, having an average return‐to‐play rate of 70.8% and recovery time of 305 days [1]. On average, 43.5% of players suffering combined ACL/MCL injuries returned to their previous level of performance. This is compared to an isolated ACL injury, which had a return‐to‐play of 74%, with 61% returning to play at least half a season [14]. The relatively small difference between isolated ACL and combined ACL/MCL injury could be due to the similarity in many combined ACL/MCL repair protocols, where the ACL is surgically reconstructed and the MCL is managed conservatively with bracing during the post‐surgical rehabilitation period [13].

Combined ACL/PCL and ACL/LCL injuries occurred equally from lateral and anterior contact to the knee, most often resulting in varus and internal rotational forces of an extended knee, while injuries of three ligaments occurred most often from anterolateral contact, causing a valgus and internal rotational force about a flexed knee, with the hip in internal rotation. Three ligament injuries exclusively occurred from direct contact. Although MLKIs all share that they are multi‐ligamentous injuries of the knee, including an ACL injury, there is significant heterogeneity in the injury patterns. Given that these injuries are rare, especially ACL/LCL, ACL/PCL and three‐ligament MLKIs, accurately characterizing MLKIs is difficult. Previous studies evaluating return‐to‐sport and return‐to‐play of MLKI in NFL athletes found a poorer prognosis among combined ACL/PCL or ACL/LCL injuries compared to ACL/MCL injury, which had an average return‐to‐play rate of 55.6%, and a recovery time of 459 days [1]. Beyond a longer recovery time, only 18.5% of athletes suffering combined ACL/LCL or ACL/PCL injury returned to their previous level of performance [1]. The worst prognosis was found in three‐ligament knee injury, with previous research identifying a mean return‐to‐play of 609 days and no players returning to their previous level of performance [1].

## LIMITATIONS

The first limitation is dependence on external sources to confirm injury, and the lack of a centralized, verified database to search for injuries. The grade of ligament injury on imaging, as well as the degree of knee laxity on examination, could not be assessed. Without imaging, it is possible that not all injuries were complete injuries, including partial ligament tears. Additionally, the lack of a centralized database leads to the chance that athletes who met the injury criteria may not have been included in the study, with higher‐profile athletes more likely to be included in the study. This may bias the result towards including more catastrophic direct injuries, which would be more likely to be within the recorded area of the field and noted by viewers than non‐contact injuries or those happening during special teams plays. However, a thorough investigation of multiple databases was utilized to minimize this concern, along with official ESPN and team injury reports to verify specific details of the injury to ensure those athletes included were categorized correctly and minimize errors of omission.

Additionally, the video clips were of variable quality and availability. Video of games from 2009 were not available on the official NFL+ service, and thus, alternative internet sources were utilized to obtain videos for analysis. Despite multiple angles, available videos did not always provide an unobstructed, zoomed‐in view of the injured knee and mechanism. Injuries that occurred with multiple nearby players or without a close view of the knee were more challenging to analyze. To account for this, all discrepancies were discussed between video reviewers to obtain consensus, and any part of the athlete that could not be accurately visualized and assessed was excluded from the analysis. Another important limitation is that the exact timing of rupture of the ligaments is unknown regarding the injury mechanism. The act of tearing a ligament is a dynamic process that occurs throughout a movement over time. While gross motions were only able to be observed with the current video analysis, small motions that were unable to be assessed likely have important implications in various injury mechanisms.

Finally, the combination of a lack of a centralized database of injury and the aforementioned limitations of the video clip limits the cohort size. As some of the injuries are relatively rare, the predominant pattern that was observed in this study may not represent the true injury mechanism, and differences between groups may or may not exist as described.

## CONCLUSION

Various injury mechanisms and characteristics result in different types of MLKI in NFL athletes, though the vast majority of MLKIs occur from direct contact forces to the knee. Combined ACL and MCL injury occurred most frequently, resembling the mechanism and lower extremity position for isolated ACL injury, though it frequently occurred due to direct contact with the knee.

## AUTHOR CONTRIBUTIONS

**Ethan Ruh**: Data collection; data analysis; manuscript writing; manuscript revision; manuscript submission. **Tyler Kallman**: Data analysis; manuscript writing; manuscript revision. **Elizabeth Lyden**: Data analysis; manuscript writing. **Justin Greiner**: Data collection; manuscript revision.

## CONFLICT OF INTEREST STATEMENT

The authors declare no conflicts of interest.

## ETHICS STATEMENT

The authors have nothing to report.

## Data Availability

The data supporting this study's findings are available from the corresponding author upon reasonable request.
